# Recognition of bird species based on spike model using bird dataset

**DOI:** 10.1016/j.dib.2020.105301

**Published:** 2020-02-20

**Authors:** Ricky Mohanty, Bandi Kumar Mallik, Sandeep Singh Solanki

**Affiliations:** a Birla Institute of Technology, Mesra, Ranchi, India; b Central Poultry Development Organisation (Eastern Region), Bhubaneswar, India

**Keywords:** Feature extraction, Classification, Recognition system, Bird species

## Abstract

Birds have often been recognised as the first informants of climatic change in our environment. Bird species recognition has assumed great significance not just for checking the survival of birds but also as an early warning signal of the declining health of earth and its climate. Earlier researchers have established recognition of bird species based on sounds from repository available online which were region-specific. In this article, we have presented the spike-based bird species recognition model, which deals with the process of identifying the bird species based on their vocalization or call. The dataset comprises of 14 bird species vocalizations. These recordings have been taken in their natural environment. The calls were recorded using a digital recorder and a unidirectional microphone at Central Poultry Development Organization (CPDO), Eastern Region, Bhubaneswar, India. The interpretation of this data provided in this article is associated with the research article titled “Automatic Bird Species Recognition System using Neural Network based on Spike" [1].

**Table:** Specifications Table

Subject	Veterinary Science, Engineering
Specific subject area	Sound recognition takes place using the audio signal of bird call
Type of data	Table -List of the 14 bird species Fig. 1(a)–(d) -Spectrogram of Japanese Quail, Aseel Brown, Guinea owl, Rhode Island Red Fig. 2 –Syllables of the Japanese Quail Fig. 3- unfiltered and filtered sound of Japanese Quail
How data were acquired	The sound recorder (Sony ICDUX560FBCE IC Audio Recorder), along with unidirectional microphone was used for collecting raw data of bird sounds. Sound Organizer is used for exchange recording files between IC recorders and computers. Intel Core i5 2010 Windows PC was used for storage of the recordings. Matlab 2015 b software was used for filtering and analysis of raw audio data.
Data format	The collection of recordings of raw bird call (audio data) in the natural environment and analysis of these data was performed.The analysis included filtering of raw data.
Parameters for data collection	The sampling frequency of bird data was 44,100 Hz where each audio recording was digitized in 16-bit accuracy. The format of the audio file was wav format.
Description of data collection	The raw data collected was registered during the day time (7 a.m.–10 a.m.) under the supervision of the veterinary doctor at the Central Poultry Development Organization, Bhubaneswar, India.
Data source location	Institution: Central Poultry Development Organization (Eastern Region) City: Bhubaneswar Country: India Latitude for collected samples: 30.694919 Longitude for collected samples:76.800275
Data accessibility	With the article Direct URL to data: https://drive.google.com/open?id=1jE2JDcq35cWRnsMoPWE6bfqhDC4Vm9TE
Related research article	Author's Name: Ricky Mohanty, Dr. Bandi Kumar Mallik, Dr. Sandeep Singh Solanki Title: Automatic Bird Species Recognition System using Neural Network based on Spike Journal: Applied Acoustics https://doi.org/10.1016/j.apacoust.2019.107177

**Table:** 

**Value of the Data** • The dataset can prove to be beneficial in further research work involving health monitoring of birds. The birds of dataset are found all over the world and very common in every part of the world. Due to the fact these birds species are commonly found, the spike based method used for identifying the species can be applicable for other species too. One of the traditional ways to detect diseased birds from the flock of healthy birds is by checking their blood sample. This way of checking the condition of birds can prove to be costly, as delay in prediction might lead to an outbreak of epidemic in birds.The prediction of disease using these birds' vocalization, would provide a different point of view for treatment. With the use of their vocalization, the recognition system can help in determining the health of the bird. The audio signal has various features that can be used for identifying and differentiating in healthy and unhealthy bird. The audio features like pitch, loudness, energy rate, zero-crossing rate, entropy, wavelength, etc. Can help detect most of the diseases caused by infection. Features like energy rate, pitch, etc. differ in case of diseased birds from the healthy birds. • As birds are essential for the world food chain, a large number of bird species can be checked using this data repository. • Birds show typical features or characteristics through their vocal signals that sometimes can indicate environmental changes

## 1 Data

The dataset in this article described in Table 1 shows the call data collected of 14 bird species from Central Poultry Development Organization, Bhubaneswar,India. In figure one, the spectrogram depicts shapes (curves) and pattern (syllable) of the birds (Japanese Quail, Guinea Fowl, Rhode Island Red and Aseel Brown). Fig. 2 illustrates the segmentation of bird sound into a series of syllables. One or more elements together make a syllable, and a set of syllables produce a phrase well explained in Fig. 2. Fig. 3 represents the filtering of Japanese quail sound.

**Table 1:** Birds found in Central Poultry Development Organization, Bhubaneswar.

SL. No.	Common name	No. of birds	Calls
1	Barred Plymouth Rock	9	50
2	White Plymouth Rock	11	39
3	Red Codish	8	40
4	Black Rock	7	28
5	Naked Neck	8	50
6	Rhode Island Red	9	46
7	Kalinga Brown	14	38
8	Japanese Quail	8	77
9	Guinea Fowl	10	41
10	Whiteleg Horn	9	36
11	AW Cross	5	27
12	Aseel Brown	15	60
13	Colour Cross	12	43
14	Vanaraja	8	65
	total	133	638

**Fig. 1:**
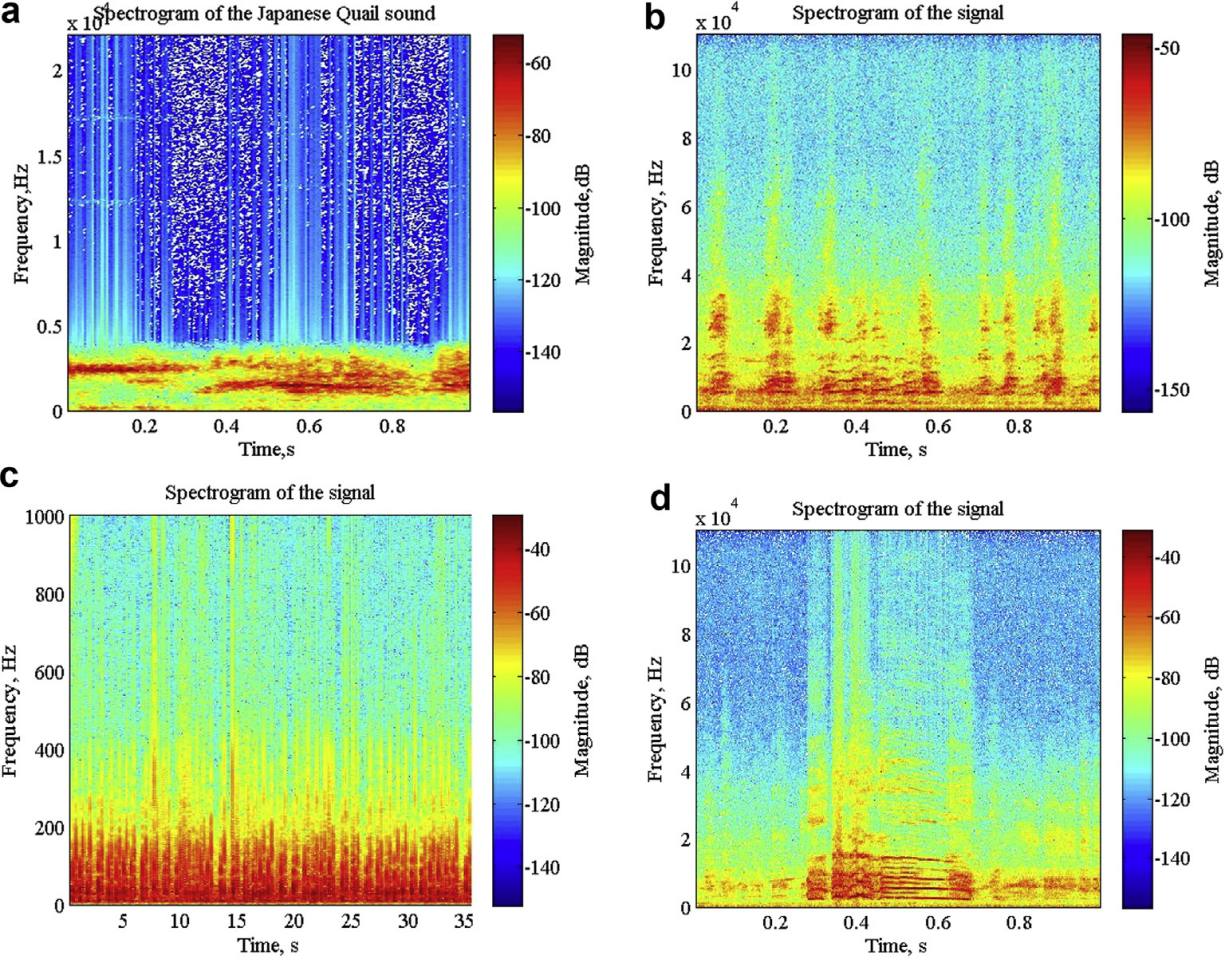
a) Spectrogram of the Japanese Quail bird sound.b) Spectrogram of the Aseel Brown bird sound. c) Spectrogram of the Guinea Fowl bird sound. d) Spectrogram of the Rhode Island Red bird sound.

**Fig. 2:**
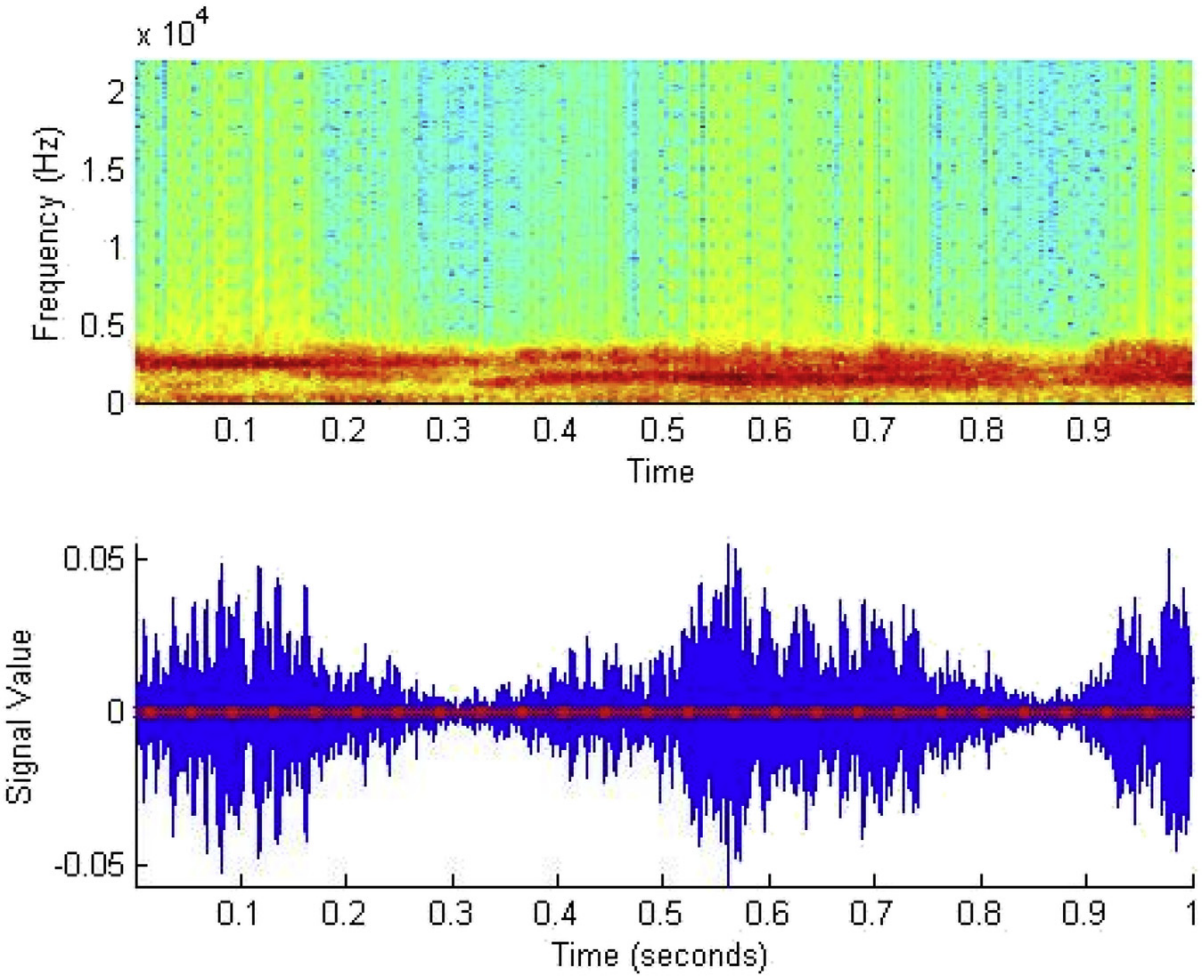
Shows the seven syllables of the Japanese Quail Bird Sound.

**Fig. 3:**
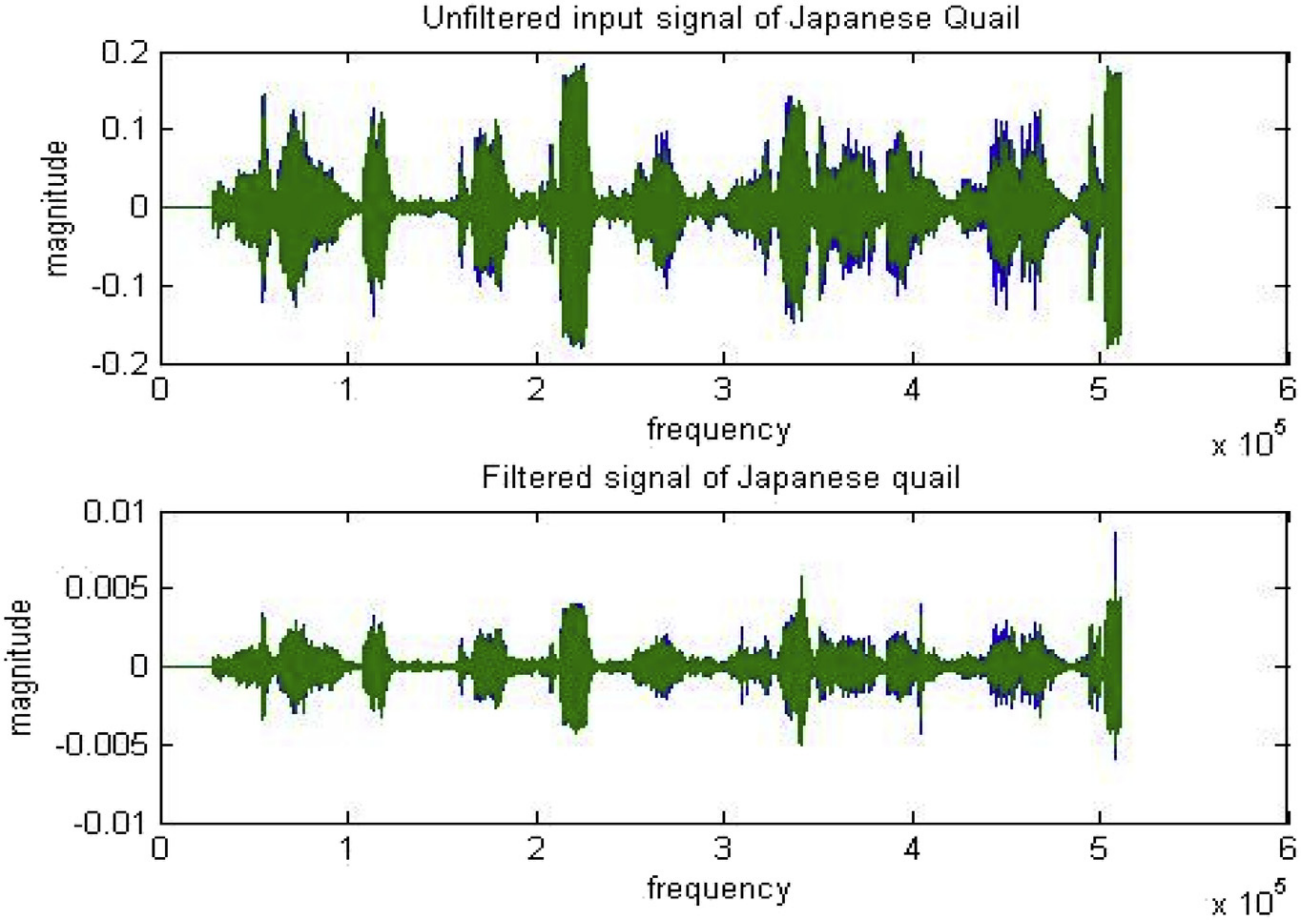
Represent the unfiltered and filtered sound of Japanese Quail.

## 2 Experimental design, materials, and methods

The automatic bird species recognition system has been designed to keep a check on a particular bird, its number, and the survival of rare species. The database collection of all species has been done using a recorder and a microphone mentioned in Table 1. These data are useful to understand the pattern of these birds, and healthy birds showed no prominent symptom of infectious disease. This data can help to investigate disease recognition for birds affected by bronchitis or respiratory infections in the future. The process started with the removal of noise as the frequency and temporal trajectories of bird sound and noise overlap. For removal of noise, a discrete wavelet transform was used as in Fig. 3.

Fig. 1(a–d) represents a pattern in the spectrogram of different bird species like Japanese Quail, Aseel Brown, Guinea Fowl, and Rhode Island Red. This pattern was used for identifying bird species as in [[5], [6], [7]]. The various works done earlier used this type of pattern of manual matching of template of the bird specific species but were less accurate in Refs. [3,4]. The shapes referred to as elements of a bird's sound.

Bird's vocalization or bird's sound split into bird call (simple and shorter sounds uttered by both sexes) and bird song (long and complicated sounds mainly uttered by male bird). The smallest part of all these birds' vocalizations is the element. So segmentation was done based on the syllable. The information contained in the syllable represents the feature vector.

For the simulation, the experiments were implemented in Matlab 2015b software. The test platform was Intel core 3i 8th generation, 2.2 GHz CPU, 4 GB RAM with Windows 10 operating system. Before the preprocessing of the raw bird sound, one-minute recording of birds was divided into 12 equal frames of 5 Sec frames by using the removal of silence Matlab code as provided in the supplementary section in Ref. [1]. Removal of noise from frames takes place using discrete wavelet transform. For the segmentation of frames, Short Time Fourier Transforms was used for obtaining spectrograms. Syllables were extracted from this spectrogram based on the energy as in Ref. [2] using syllable segmentation Matlab code as provided in the supplementary section [1]. Syllables were used for feature extraction, and then the standardization process of features takes place. This standardization was followed by the classification and recognition of bird species using the Spiking Neural Network with the Permutation Pair Frequency Matrix Matlab code as provided in Ref. [1].
